# Comparative Efficacy and Safety of Ketamine Versus Midazolam for Suicidality: A GRADE‐Assessed Systematic Review and Meta‐Analysis of Randomized Controlled Trials

**DOI:** 10.1002/brb3.71255

**Published:** 2026-02-08

**Authors:** Asim Shah, F. N. U Sawaira, Misbahuddin, Muneeb Shad Mohmand, Suleman Khan, Zaryab Bacha, Hammad Iftikhar, Asad Jamal, Fazia Khattak, Aizaz Anwar Khalid, Aamer Syed, Kamil Ahmad Kamil

**Affiliations:** ^1^ Khyber Medical College Peshawar Pakistan; ^2^ Khyber Girls Medical College Peshawar Pakistan; ^3^ Peshawar Medical College Peshawar Pakistan; ^4^ Internal Medicine Department Mirwais Regional Hospital Kandahar Afghanistan

**Keywords:** depression, ketamine, meta‐analysis, midazolam, randomized controlled trials, rapid‐acting antidepressants, suicidal ideation

## Abstract

**Background:**

Acute suicidality is an emergency psychiatric condition that requires urgent treatment. Traditional treatment approaches, such as antidepressant pharmacotherapy and psychotherapy, take weeks to achieve optimal effectiveness, hence exposing patients in imminent crisis to significant risk. Ketamine is a fast‐acting N‐methyl‐D‐aspartate (NMDA) receptor antagonist that has been suggested as a possible treatment option for suicidality. The current meta‐analysis aims at comparing the relative efficacy and safety of ketamine with midazolam, an active sedative/anxiolytic comparator used in ketamine trials to support masking/blinding and control nonspecific acute effects, for reducing suicidal ideation and co‐occurring depression severity.

**Methods:**

The systematic search was done on PubMed, Embase, and Cochrane Central databases by July 2025. Randomized controlled trials (RCTs) that involved ketamine and midazolam in adults with acute suicidality were chosen. The major outcomes were the variations in suicidal ideation as assessed by the Montgomery–Åsberg Depression Rating Scale—Suicidal Ideation item (MADRS‐SI) and Beck Scale of Suicide Ideation (BSS). Additional outcomes were the severity of depression in general (Montgomery–Åsberg Depression Rating Scale (MADRS) total score) and adverse events. Pooled effects were calculated using a random‐effects model.

**Results:**

This systematic review comprised ten randomized controlled trials (RCTs) with 649 participants. The meta‐analysis showed that the administration of ketamine was linked to a considerable decrease in suicidal ideation in comparison to midazolam with mean differences of −1.23 points on the Montgomery–Åsberg Depression Rating Scale (MADRS‐SI; 95% confidence interval (CI) −2.14 to −0.32) and −4.30 points on the Beck Scale for Suicide Ideation (BSS; 95% CI −8.01 to −0.59). Ketamine was also associated with reduced severity of depressive symptoms compared to midazolam, with a difference of −6.23 (95% CI −10.37 to −2.08) on the MADRS total score. At the same time, adverse events such as nausea, emotional disturbance, derealization, and dizziness were much more frequent in the ketamine group.

**Conclusions:**

Ketamine is much more effective than midazolam in the short‐term improvement of suicidal ideation and depressive symptoms in adult patients with acute suicidality. Despite the fact that ketamine is linked to increased rates of transient adverse effects, its quick action makes it an especially appealing intervention in emergency psychiatric treatment. Further research is thus needed to clarify the long‐term effectiveness of the agent and to optimize the best treatment regimes.

## Graphical abstract

Ketamine rapidly reduced suicidal ideation and depressive symptom severity more than midazolam in adults with acute suicidality across 10 RCTs (*n* = 649). Transient adverse effects (e.g., nausea, derealization, dizziness) were more frequent with ketamine, supporting its use as a fast‐acting emergency intervention with safety monitoring.

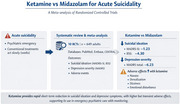

## 1 Introduction

Suicidal behavior represents a complex, urgent psychiatric crisis that necessitates immediate, effective action. In the United States, suicide is one of the leading causes of death and is considered largely preventable. (Suicide—National Institute of Mental Health (NIMH)) Globally, suicide is a major public health problem and one of the leading causes of preventable death, with the World Health Organization (WHO) estimating that more than 700,000 people die by suicide each year (Suicide World Health Organization (WHO)). The acute suicidality, especially in individuals with major depressive disorder (MDD) or other mood disorders, is thus a serious public health issue (Cai et al. 2021).

The existing first‐line interventions for suicidality, which are primarily antidepressant pharmacotherapy and psychotherapy, may take weeks to show any real improvement (Nischal et al. 2012). This delay in time is a problem for individuals who are at imminent risk (Bachmann 2018). Ketamine, a glutamatergic NMDA receptor antagonist, is becoming increasingly recognized as a rapid‐acting antidepressant (Krystal et al. 2013). According to several studies, ketamine has the ability to reduce suicidal ideation within a few hours after its administration (Zarate et al. 2006).

Despite the recognition of the usefulness of ketamine in the treatment of acute suicidality, the degree of its superiority compared with other active interventions is not clear (Dadiomov and Lee 2019). Midazolam, a short‐acting benzodiazepine used commonly as an anxiolytic and sedative in acute situations (Peter et al. 2024), is often used as a comparator in ketamine studies (Wilkinson et al. 2019).

These two drugs have a different mechanism of action. The main mechanism of action of ketamine is the NMDA (N‐methyl‐D‐aspartate) receptor antagonist (Iadarola et al. 2015). This causes alterations in the brain's glutamatergic signaling. Ketamine inhibits NMDA receptors, which decrease excitatory neurotransmission. This facilitates the greater secretion of brain‐derived neurotrophic factor (BDNF) and provokes synaptogenesis (the creation of new synaptic connections) (Mion and Villevieille 2013; Abdallah et al. 2016). This process is believed to help in the quick antidepressant action of ketamine, which is usually experienced in hours.

Conversely, midazolam targets the gamma‐aminobutyric acid type A (GABA‐A) receptor. Midazolam decreases the excitability of the neurons by increasing the inhibitory action of GABA (Wang et al. 2020). This causes sedation, anxiolysis, and muscle relaxation, making it beneficial in the treatment of agitation or acute anxiety. Nevertheless, midazolam fails to treat neurobiological causes of depression and suicidality in the same manner as ketamine (Murrough et al. 2013).

The other important distinction between ketamine and midazolam is the duration of action. The effects of ketamine are quick but are normally short‐lived, and they may last a few hours to a few days following a single infusion (Hochschild et al. 2022). This is a temporary effect that may require repeated doses to be beneficial. Midazolam, on the other hand, offers temporary relief for a few hours. Midazolam is a short‐acting benzodiazepine with sedative/anxiolytic effects and is used in ketamine trials as an active (psychoactive) comparator to help preserve blinding and control for nonspecific acute effects. Importantly, midazolam is not an antidepressant and is not an established treatment for suicidality; therefore, it serves as a control condition rather than a therapeutic intervention for depression or suicidal ideation. (Barzkar et al. 2025).

Meta‐analyses of the efficacy of ketamine in suicidality often combine different comparators, including saline or midazolam, with no obvious distinction (Wilkinson et al. 2019; Sajid et al. 2024; Wilkinson et al. 2018). This may complicate the assessment and interpretation of ketamine's true effectiveness, as these factors influence how suicidality outcomes are measured and reported across trials. To fill this gap, the current meta‐analysis will compare ketamine and midazolam on acute suicidality and depression severity. This paper will focus on randomized controlled trials (RCTs) that directly compare the two treatments, providing a more precise estimate of their comparative efficacy.

Although ketamine's rapid effects on suicidal ideation have been described in broader reviews that pooled heterogeneous comparators, the primary aim of the present study was to address a narrower, clinically focused question: how ketamine compares with an active sedative/anxiolytic control (midazolam) for acute suicidality under blinded randomized conditions. By restricting inclusion to head‐to‐head randomized controlled trials using midazolam, we sought to minimize expectancy effects and nonspecific sedation‐related improvements and to provide a comparator‐specific synthesis integrating efficacy, safety outcomes, and certainty of evidence using the GRADE framework. We will evaluate important outcomes, such as suicidal ideation, which will be evaluated with the help of the Montgomery–Åsberg Depression Rating Scale (MADRS‐SI) and the Beck Scale of Suicide Ideation (BSS). The Montgomery–Åsberg Depression Rating Scale (MADRS) will be used to measure the severity of depression. We will also compare the adverse event profiles of the two treatments to assess the safety profiles of the two treatments. This will give a clear evaluation of their clinical usefulness in the treatment of acute suicidality.

## 2 Methods

### 2.1 Study Protocol and Registration

This meta‐analysis adhered to the guidelines outlined in the Cochrane Handbook for Systematic Reviews and Interventions and the Preferred Reporting Items for Systematic Reviews and Meta‐Analyses (PRISMA) guidelines 2020 (The PRISMA 2020 statement: an updated guideline for reporting systematic reviews). The protocol for this meta‐analysis was registered with PROSPERO (CRD420251110292).

### 2.2 Search Strategy and Databases

PubMed, Embase, and Cochrane Central were electronically searched without date restrictions up to July 2025. Our search was not limited by language in English and was done using the following search terms: ketamine, suicidal ideation, suicide attempts, midazolam, depression, and treatment‐resistant depression. A thorough search of all databases with Mesh words (ketamine) AND (suicide OR suicidality OR “suicidal ideation” OR “suicidal thoughts” OR “suicide attempts”) AND (midazolam OR depression OR depressive OR “major depressive disorder” OR MDD). Additionally, the reference lists of the included studies and related systematic reviews were manually searched to identify other relevant studies.

### 2.3 Eligibility Criteria

Studies were included based on the following PICO criteria:

- **Population (P)**: Adults with acute suicidality or suicidal ideation, regardless of psychiatric diagnosis
- **Intervention (I)**: Ketamine (administered via any route: intravenous, intranasal, or oral)
- **Comparator (C)**: Midazolam, as an active (psychoactive) placebo comparator used to enhance masking/blinding
- **Outcomes (O)**:
  ○ **Primary**: Suicidal ideation, measured by MADRS‐SI (Montgomery–Åsberg Depression Rating Scale—Suicidal Ideation item) and BSS (Beck Scale for Suicide Ideation)
  ○ **Secondary**: Depression severity, measured by MADRS (Montgomery–Åsberg Depression Rating Scale total score)
  ○ **Safety**: Adverse events (e.g., dissociative symptoms, anxiety, sedation)

The scales' definitions are given in the supplementary materials.

- **Study Type**: Randomized controlled trials (RCTs)

We excluded studies based on the following criteria:

- Studies that did not report relevant outcomes (e.g., suicidality, depression severity, adverse events)
- Studies not comparing ketamine to midazolam
- Non‐randomized studies (e.g., cohort studies, case series, observational studies)
- Review articles, meta‐analyses, case reports, conference abstracts, animal studies, and studies not published in English

### 2.4 Study Selection

The databases were used to retrieve studies that were subsequently imported into the Rayyan software for screening. Duplicates were deleted automatically. The titles and abstracts were screened by two independent investigators (A. S. and M. U.). The inclusion criteria were used to review the full texts of potentially eligible studies. The third investigator resolved disagreements between reviewers (SK). Screening resulted in 10 randomized controlled trials. The PRISMA flow diagram (Figure S1 **in the Supplementary Material**) summarizes the process of selecting the studies.

### 2.5 Data Extraction

Four investigators (A. S., M. U., S. K., and A. J.) independently extracted relevant data from the included studies. The following details were extracted:

- **Study characteristics**: Author(s), publication year, sample size, country, study design
- **Intervention details**: Dosage and route of administration for ketamine and midazolam, duration of treatment
- **Participant characteristics**: Age, sex, baseline suicidality severity, and baseline depressive symptom severity (depression severity score on a validated scale, e.g., MADRS).
- **Outcome measures**: Suicidal ideation (measured by MADRS‐SI and BSS), depression severity (measured by MADRS), and adverse events (including dissociative symptoms, sedation, and anxiety)

Any discrepancies in the data extraction process were resolved through discussion, and a final consensus was reached. Data were also cross‐checked for consistency.

### 2.6 Type of Interventions

In the included trials, ketamine was administered predominantly by intravenous infusion at subanesthetic doses (most commonly 0.5 mg/kg infused over ∼40 min). One included trial administered oral racemic ketamine. Midazolam served as the active control and was administered via the corresponding route.

### 2.7 Risk of Bias Assessment

We assessed the risk of bias in the included studies using the Cochrane Risk of Bias Tool. The following domains were evaluated for each trial:

- Random sequence generation
- Allocation concealment
- Blinding of participants and outcome assessors
- Incomplete outcome data
- Selective reporting

All studies included in the meta‐analysis were found to have a low risk of bias (ROB) across all domains, as they adhered to rigorous methods in randomization, allocation concealment, and blinding. **(ROB plots are given in the Supplementary** Figure **S2** **.)**

### 2.8 Statistical Analysis

The analysis of the data was conducted using RevMan Version 5.4 software, which employs a random‐effects model. Measures of effect were as follows: Mean Difference (MD) in cases of continuous outcomes (e.g., Suicidal ideation and depression severity, measured by MADRS‐SI and BSS). Odd Ratio (OR) of binary outcomes (e.g., response rates of suicidality). We evaluated the heterogeneity among the studies through the I^2^ statistics, where values greater than 50% indicated a significant heterogeneity. In case of significant heterogeneity (*I* ^2^ > 50%), a leave‐one‐out sensitivity analysis was performed by removing studies one at a time to investigate their impact on the overall findings. All the statistical tests were two‐tailed, and a *p*‐value of < 0.05 was significant.

### 2.9 Study Characteristics

The 10 RCTs, which were carried out in 2013–2025, involved a total of adult participants with major depressive disorder, treatment‐resistant depression, or acute suicidal ideation. The number of participants in the samples ranged from 20 to 84. The research was conducted in various locations, including psychiatric inpatient units, outpatient clinics, and tertiary care hospitals, across North America, Europe, and Asia. The most frequent route of administration of ketamine was intravenous at a subanesthetic dose of 0.5 mg/kg, while one included trial used oral racemic ketamine. Midazolam, usually administered intravenously at anxiolytic doses, was used as an active control to increase blinding. In the majority of studies, the intervention consisted of a single infusion, whereas others involved repeated dosing over 1 to 2 weeks. Outcome measures were validated scales of suicidal ideation (Montgomery–Åsberg Depression Rating Scale—Suicidal Ideation item (MADRS‐SI), Beck Scale for Suicide Ideation (BSS), and Scale for Suicide Ideation (SSI)) and depression severity (Montgomery–Åsberg Depression Rating Scale (MADRS) and Hamilton Depression Rating Scale (HDRS)). The follow‐up was conducted between 240 min after infusion and 14 days after infusion, allowing for the evaluation of the immediate effects of ketamine. The baseline characteristics of age, gender, and severity of depression were well‐balanced in general between treatment arms, which is favorable to the internal validity of the pooled analysis. The study and baseline characteristics are presented in **Supplementary Tables** **1** **and** **2** **in the Supplementary Material**.

## 3 Results

A search of databases yielded 1349 records. One thousand and twenty records were screened after removing 329 duplicates. Out of these, 940 were excluded because they failed to meet the inclusion criteria. Eighty reports were reviewed in full text, and 70 were eliminated due to inappropriate comparisons or results. Ten studies were identified as meeting the inclusion criteria; thus, they were included in further meta‐analysis.

### 3.1 Primary Outcomes

#### 3.1.1 Montgomery–Åsberg Depression Rating Scale—Suicidal Ideation (MADRS‐SI)

**FIGURE 1:**
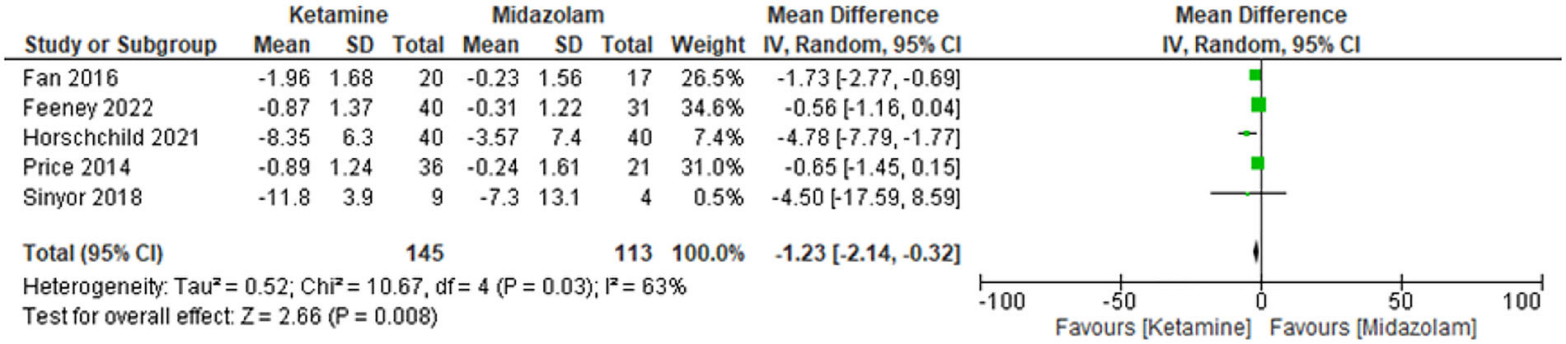
Forest plot MADRS‐SI: Ketamine vs. midazolam for suicidal ideation; pooled mean difference with 95% CI, random‐effects.

Ketamine also indicated a significant decrease in suicidal ideation when compared with midazolam. The pooled mean difference of MADRS‐SI was −1.23 (95% confidence interval [CI]: −2.14 to −0.32), with a *p*‐value of 0.008, indicating that ketamine was more effective in ameliorating suicidal ideation. The I^2^ heterogeneity of the studies was moderate (*I* ^2^ = 63%), indicating some variation in effect sizes between studies. Exclusion of the study by Hoschchild et al. (2021) as part of the sensitivity analysis did not have a significant impact on the overall effect size. The MD was −0.86 (95% CI: −1.42 to −0.29), *p*‐value = 0.003 after exclusion. The heterogeneity was reduced to 27%, meaning that the omitted study contributed some value to the results. These results stand to confirm the effectiveness of the ketamine effect in the decrease of suicidal ideation (Figure **1**).

#### 3.1.2 Beck Scale for Suicide Ideation (BSS)

**FIGURE 2:**
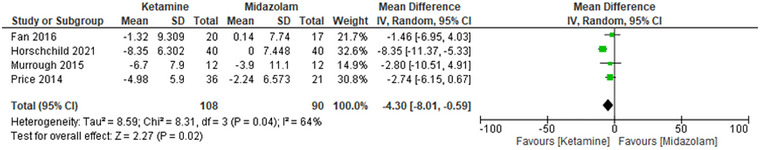
Forest plot BSS: Ketamine vs. midazolam for suicidal ideation; pooled mean difference with 95% CI, random‐effects.

Ketamine showed a tremendous decrease in suicidal thoughts compared to midazolam as evaluated by BSS. The pooled Mean Difference was −4.30 (95% CI: −8.01 to −0.59), and the *p*‐value of this figure was 0.02. This outcome was heterogeneous, with a low value (*I* ^2^ = 64%). The overall effect was not affected by sensitivity analysis excluding Hoschchild et al. 2021. The MD has taken the same value as before, or −2.44 (95% CI: −5.15 to −0.28), and the *p*‐value came down to 0.08 with the heterogeneity dropping to 0%, which once again showed that the omitted study was one of the sources of the inconsistency in the findings. These statistics support and confirm the conclusion that ketamine is much superior to midazolam about improvement of suicidal ideation as per the BSS (Figure **2**).

### 3.2 Secondary Outcomes

#### 3.2.1 Montgomery–Åsberg Depression Rating Scale (MADRS)

**FIGURE 3:**
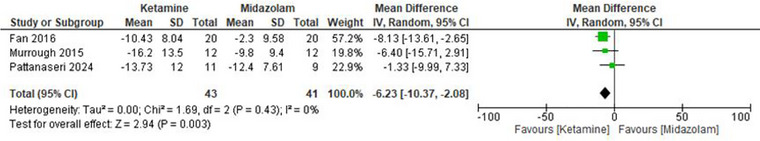
Forest plot MADRS total: Ketamine vs. midazolam for depression severity; pooled mean difference with 95% CI, random‐effects.

There was a notable improvement in the severity of depression as estimated using MADRS in ketamine. The pooled Mean Difference turned out to be −6.23 (95% CI: −10.37 to −2.08), with a p‐value of 0.003, indicating that ketamine is more effective than midazolam in relieving depressive symptoms. There was an extremely low heterogeneity (*I* ^2^ = 0%). These results underline the large antidepressant impact of ketamine on this group (**Figure** **3**).

### 3.3 Safety Outcomes

#### 3.3.1 Nausea and Vomiting

**FIGURE 4:**
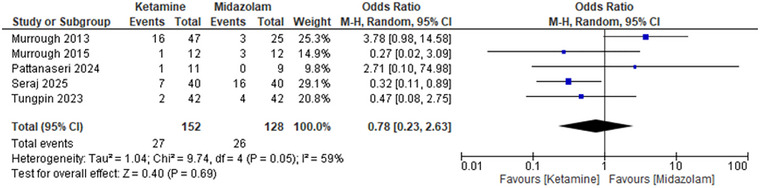
Forest plot nausea and vomiting: Pooled odds ratio with 95% CI, random‐effects.

Ketamine was associated with a lower incidence of nausea and vomiting compared to midazolam. The pooled Odds Ratio for nausea/vomiting was 0.78 (95% CI: 0.23 to 2.63), with a *p*‐value of 0.69, suggesting a lower risk of these adverse events in individuals receiving ketamine. However, this difference did not reach statistical significance. The level of heterogeneity was moderate (59%). Sensitivity analysis of the exclusion of the study by Murrough et al. (2013) indicated that the lower incidence of nausea/vomiting was now significant, with an OR of 0.39 (95% CI: 0.17 to 0.87) and a *p*‐value of 0.02. The heterogeneity decreased to 0%, which means that the study contributed to a certain variability in the data. These findings suggest that ketamine is associated with a lower incidence of nausea and vomiting (Figure 4).

#### 3.3.2 Emotional Disturbances

**FIGURE 5:**
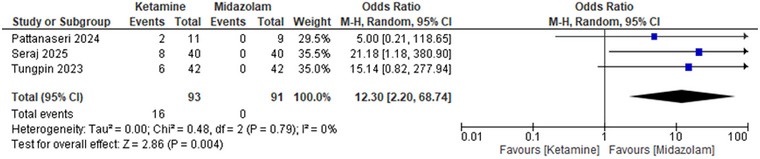
Forest plot emotional disturbances: Pooled odds ratio with 95% CI, random‐effects.

Emotional disturbances based on the outcome of using ketamine were much more likely to occur as a result of using ketamine than midazolam, with an Odds Ratio of 12.30 (95% CI: 2.20 to 68.74) and a *p*‐value of 0.004. The heterogeneity equaled 0, which indicated the high side effect of ketamine (Figure 5 **)**.

#### 3.3.3 Derealization

**FIGURE 6:**
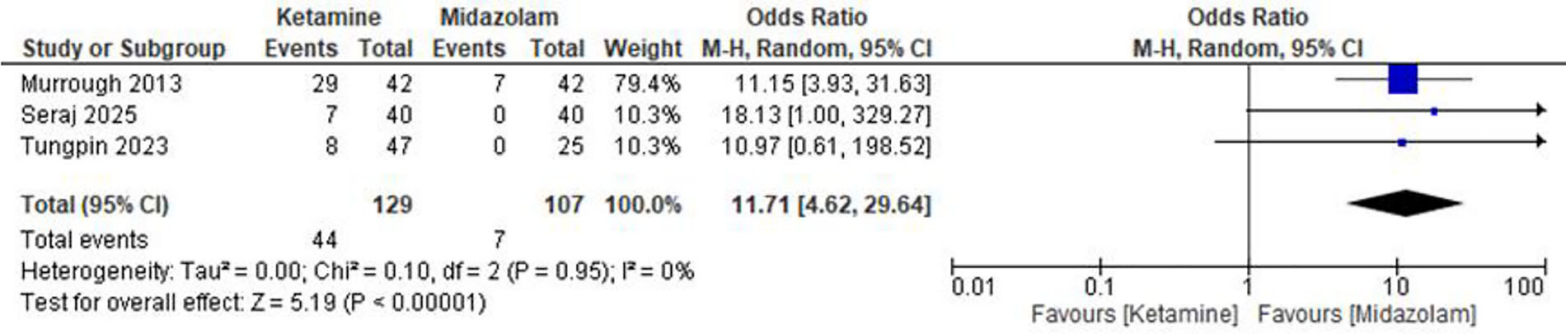
Forest plot derealization: Pooled odds ratio with 95% CI, random‐effects.

Ketamine was found to have a significantly higher occurrence of symptoms of derealization (*p*‐value of 0.00001) than midazolam, with an Odds Ratio of 11.71 (95% CI: 4.62 to 29.64), indicating that ketamine was more prone to cause derealization compared to midazolam. The heterogeneity was quite small (*I* ^2^ = 0%), indicating that the data were slightly different. These findings highlight the elevated likelihood of derealization during the use of ketamine (Figure 6).

#### 3.3.4 Dizziness

**FIGURE 7:**
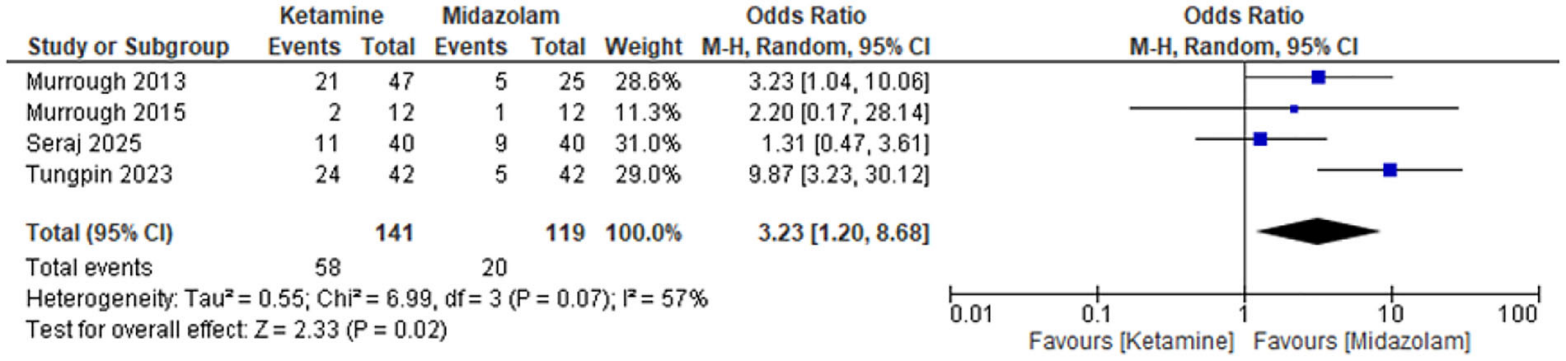
Forest plot dizziness: Pooled odds ratio with 95% CI, random‐effects.

The chances of dizziness were higher with ketamine compared to midazolam. The pooled Odd Ratio before sensitivity analysis was 3.23 (95% CI: 1.20 to 8.68), *p*‐value 0.02, and heterogeneity 57%. After sensitivity analysis by the removal of the study by Tungpin 2023, the OR became 1.97 (95% CI: 0.95 to 4.08), with a *p*‐value 0.07, which shows that ketamine is associated with an increased incidence of dizziness compared to midazolam. This observation highlights dizziness as one of the common side effects of ketamine (Figure 7).

**(Before and after sensitivity analysis, forest plots are given in the Supplementary Material.)**

The confidence of evidence for primary outcomes (MADRS‐SI and BSS) was moderate and was downgraded due to inconsistency. The severity of depression (MADRS) was also of moderate certainty, mainly because of imprecision. Derealization was characterized by high‐certainty evidence among adverse events, whereas nausea, dizziness, and emotional disturbances were supported by moderate‐certainty evidence. **The full GRADE Summary of findings is provided in Supplementary** **Table** **S3**.

## 4 Discussion

This meta‐analysis affirms the fact that ketamine is more effective than midazolam in mitigating suicidal ideation and depressive symptoms among adults with acute suicidality. The size of the effect on validated scales such as the MADRS‐SI, the BSS, and the MADRS total score indicates that ketamine is associated with clinically meaningful short‐term improvements in suicidal ideation and depressive symptoms compared with midazolam. Notably, the effects remained similar even following sensitivity analysis, which further supported the importance of these findings. Across the included trials, which enrolled high‐risk clinical populations with primary psychiatric disorders, medical comorbidities, or both, ketamine demonstrated superiority over midazolam in reducing suicidal ideation and depressive symptoms. Importantly, this review is intended as a rigorous, comparator‐specific synthesis rather than a claim of a novel efficacy signal. The evidence base for ketamine versus midazolam remains modest, and several pooled estimates necessarily include small numbers of studies after sensitivity analyses to explore heterogeneity. Presenting these findings alongside GRADE certainty ratings is intended to help clinicians and guideline developers interpret the strength and limitations of the current evidence for this specific active‐controlled comparison.

In comparison with the rest of the existing body of literature, these findings not only confirm but also improve the earlier conclusions. Many studies have confirmed the ability of ketamine to reduce suicidal thoughts within hours of use (Wilkinson et al. 2018; Abbar et al. 2022; Hochschild et al. 2021). Prior studies have noted that differences in control conditions and the potential for unblinding/expectancy effects can influence estimated effect sizes; therefore, interpretation of the magnitude and clinical relevance of ketamine's short‐term effects may vary across the literature (Wilkinson et al. 2019; Wilkinson et al. 2018; Hochschild et al. 2021; Seyedoshohadaei et al. 2024). In comparison, the present meta‐analysis is limited to the studies that involved midazolam as an active comparator, replicating the psychoactive and sedative effects of ketamine but not its antidepressant effects. This difference is significant, as midazolam assists in maintaining blinding and managing nonspecific treatment effects, that is, sedation, anxiolysis, and altered consciousness (Tabari et al. 2025).

It has also been argued in the literature whether the anti‐suicidal effect of ketamine is independent of its antidepressant properties. There is some evidence that decreases in suicidal ideation are closely associated with mood changes, which may indicate a downstream effect of general symptom improvement (Price and Mathew 2015; Matveychuk et al. 2020). Some people say that ketamine can have direct anti‐suicidal effects through different mechanisms, including the modulation of glutamate signaling, synaptic plasticity, and neural connectivity in circuits involved in hopelessness and emotional pain (Krystal et al. 2023). This analysis partially supports the view that reductions in suicidal ideation may occur alongside improvements in depressive symptom severity, as decreases in suicidal thoughts were observed in parallel with reductions in depression severity. Nevertheless, the observation that ketamine was superior to midazolam in both measures, even in short‐term measures, indicates a more complicated interaction between the immediate improvement of mood and the specific alleviation of suicidal thinking.

Moreover, the current literature has revealed disparities in the durability of ketamine's effects on suicidality and depressive symptoms. Some studies report that the antisuicidal and antidepressant effects attenuate within a few days, whereas others indicate that repeated dosing may prolong these clinical effects for several weeks (Sakopoulos and Todman 2025; McMullen et al. 2021). In the current analysis, the majority of the studies reported results within 7 days, which demonstrates a high short‐term efficacy, and the long‐term durability remains unanswered (Glue et al. 2024). However, because the included trials assessed outcomes at different follow‐up times (ranging from 24 h to approximately 1 month), this meta‐analysis was not designed to determine the precise onset of ketamine's effect. This limitation mirrors the broader ketamine literature beyond midazolam‐controlled RCTs, in which dosing schedules (single vs. repeated infusions), maintenance strategies, adjunctive treatments, and follow‐up time points vary across studies. Also, though prior reviews have observed the possibility of rapid relapse following initial response, the short‐term efficacy demonstrated here provides further support for the use of ketamine as an intervention in suicidal crises, while underscoring the need for appropriate long‐term management strategies (Jobnah et al. 2024; Kritzer et al. 2022).

Tolerability is another vital factor. There is a general consensus in the literature that the dissociative and perceptual effects of ketamine may be disturbing to some patients, particularly those with a trauma history or with psychotic characteristics (Correia‐Melo et al. 2017; Ballard and Zarate 2020). We have found that the adverse effects are more frequent than those of midazolam, especially dizziness, derealization, and emotional disturbances. These side effects are mostly mild to moderate and temporary in nature, but they are a significant consideration in risk‐benefit analysis. Of particular significance, midazolam controls have been instrumental in demonstrating that numerous adverse effects that are often ascribed to ketamine are not observed with structurally similar sedatives, which highlights their drug specificity (Sener et al. 2011).

The most studied and effective route of administration is intravenous ketamine. However, recent research has considered the application of intranasal and oral preparations because they are easy to administer and more accessible (Dasari et al. 2024). Although IV ketamine is the most studied route, the trials included in this meta‐analysis were predominantly IV, with one oral trial; therefore, conclusions about comparative efficacy by route are limited. This is consistent with the existing standards of clinical practice and the lack of further comparative research on routes of administration, especially in outpatient and resource‐constrained settings.

This meta‐analysis demonstrated the various strengths of the quality of evidence, as assessed by the GRADE method. The majority of the outcomes received a moderate certainty rating, indicating that they are reliable in informing clinical decisions. It is essential to note that the result of derealization was supported by high‐certainty evidence, indicating consistency in the results of studies with high data integrity. Moreover, the risk of bias in the included studies was generally low, and few concerns were raised regarding indirectness or publication bias, which enhances the internal validity of the findings.

Nevertheless, a few limitations are worth noting. One of the concerns was inconsistency, or heterogeneity, which was observed in several outcomes, including suicidal ideation (MADRS‐SI and BSS), nausea, and dizziness, and resulted in a downgrade in the certainty ratings. Also, the estimates of depression severity (MADRS) and emotional disturbances were imprecise, primarily because of small sample sizes and large confidence intervals, which restrict the precision and generalizability of the results. The other major weakness was the limited follow‐up period of the majority of the trials (≤14 days), which limited the conclusions on the long‐term efficacy and safety of ketamine.

To summarize, the certainty of the evidence regarding the superiority of ketamine over midazolam in the treatment of acute suicidality and depression is moderate, and one outcome, derealization, has high‐certainty evidence. This implies that although ketamine is a potential intervention in acute psychiatric care, especially when it comes to providing rapid symptomatic relief, additional high‐quality studies are required to shed some light on its long‐term efficacy, adverse event profiles, and ideal treatment regimens. Ketamine may be considered a potential short‐term strategy for the acute management of suicidality, provided that its use is accompanied by careful monitoring and an individualized risk‐benefit assessment.

On the whole, the findings of this meta‐analysis are consistent with the existing literature in showing the swiftness of ketamine in treating suicidality and depression. Nevertheless, this analysis is more conservative and arguably more clinically relevant since it only considers active‐controlled trials with midazolam. It also fills a major gap in the literature by offering a direct comparator that isolates the pharmacologic effects of ketamine from the general sedative effect. Notably, one included midazolam‐controlled inpatient pilot trial was terminated early and therefore contributes limited precision (Sinyor et al. 2018)_._

## 5 Conclusion

This meta‐analysis shows that ketamine is much more effective than midazolam in the immediate improvement of suicidal ideation and the severity of depression in adults with acute suicidality. It is a useful intervention in psychiatric crises because of its rapid antidepressant effects, which are apparent within hours. Although ketamine has a greater risk of temporary side effects, it is more advantageous than midazolam, which does not have antidepressant effects. These results justify the clinical application of ketamine in acute care, and additional studies are necessary to determine long‐term effectiveness and the best treatment regimens.

## Author Contributions

A. S. contributed to conceptualization, supervision, and project administration. AS and SK were responsible for data curation, resources, and software. F. S., M. S. M., and A. J. contributed to writing – review and editing, and validation. F. S. was involved in validation and writing – review and editing. M. U. and H. I. participated in the investigation, writing the original draft, and visualization. A. A. K. and A. Sy. contributed a the formal analysis and investigation. Z. B. contributed to methodology and formal analysis. A. S. and M. U. were involved in data interpretation, final manuscript drafting, and editing. All authors have read and approved the final version of the manuscript.

## Funding

The authors have nothing to report.

## Conflicts of Interest

The authors declare no conflicts of interest.

## Supporting information




**Supplementary Materials**: brb371255‐sup‐0001‐SuppMat.docx

## Data Availability

The data supporting the findings of this study are available from the corresponding author upon reasonable request.
