# Supplementary material for: Comparative Efficacy and Safety of Ketamine Versus Midazolam for Suicidality: A GRADE‐Assessed Systematic Review and Meta‐Analysis of Randomized Controlled Trials
Source: Brain Behav. 2026 Feb 8;16(2):e71255. doi: 10.1002/brb3.71255 (PMC12883703; doi:10.1002/brb3.71255)
Supplement: Supplementary file 1 — Supplementary Materials: brb371255‐sup‐0001‐SuppMat.docx [file BRB3-16-e71255-s001.docx]

**Supplementary Files**

**Scales Definition:**

- MADRS-SI (Montgomery–Åsberg Depression Rating Scale – Suicidal Ideation item): A single item from the MADRS that specifically assesses the severity of suicidal thoughts, rated on a 0–6 scale.

- MADRS (Montgomery–Åsberg Depression Rating Scale):

A 10-item clinician-rated scale used to assess overall depression severity. Each item is scored 0–6, with higher scores indicating more severe symptoms.

- BSS (Beck Scale for Suicide Ideation):

A 21-item self-report or clinician-administered scale that measures the intensity, duration, and frequency of suicidal thoughts and intentions. Each item is rated 0–2.

**(Figures)**

**Figure 1**

**Identification of studies via databases and registers**

Records removed *before screening*:

Duplicate records removed

(n =329)

Records identified from*:

Databases (n = 1349)

PubMed = 896

Embase = 128

Cochrane = 325

**Identification**

Records screened

(n = 1020)

Records excluded**

(n = 940)

Reports sought for retrieval

(n = 80)

Reports not retrieved

(n = 0)

**Screening**

Reports assessed for eligibility

(n = 80)

Reports excluded:

Reason 1 (n = 15)

Reason 2 (n = 40)

Reason 3 (n =15)

Studies included in review

(n = 10)

Reports of included studies

(n =10)

**Included**

- Records excluded by Primary Screening (n = 940)

Reason-1: Not ketamine vs midazolam

Reason-2: Different Intervention and Control arm

Reason-3: Did not report relevant outcomes

Source: Page MJ, et al. BMJ 2021;372:n71. doi: 10.1136/bmj.n71.

This work is licensed under CC BY 4.0. To view a copy of this license, visit <https://creativecommons.org/licenses/by/4.0/>

**Figure 2**

1. **Risk of Bias Assessment Figures:**
2. **Traffic Light Plot:**


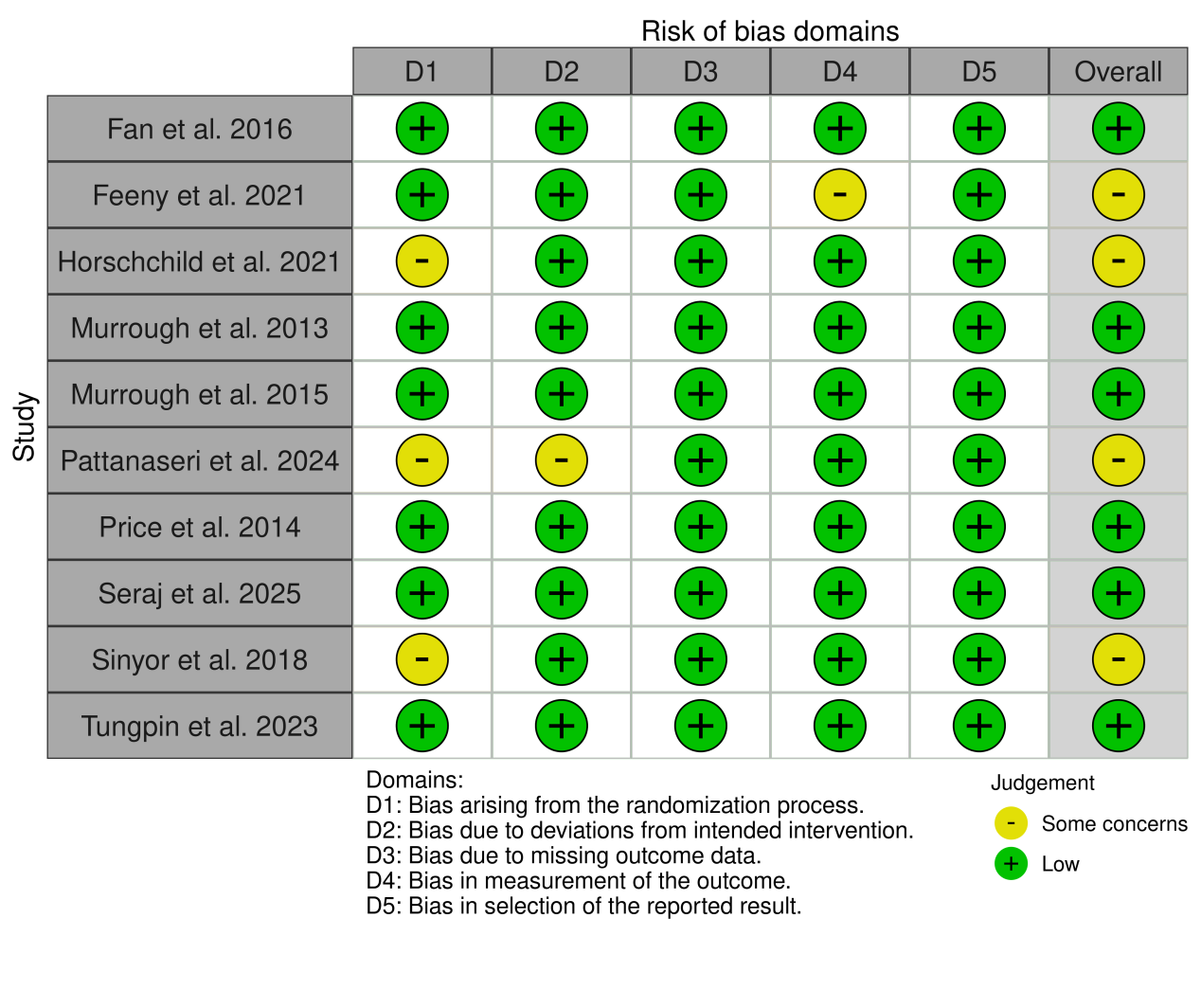


1. **Summary plots:**


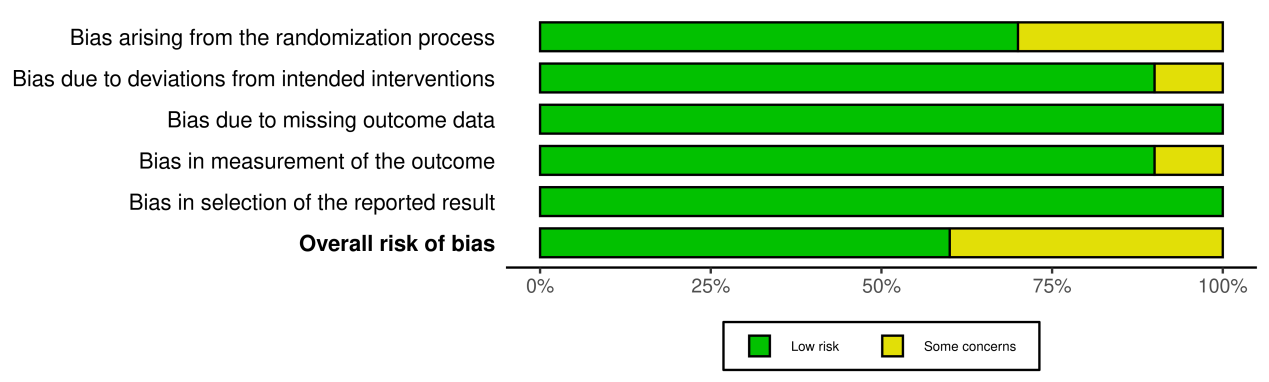


**(Tables)**

**Supplementary Table 1.** Study characteristics of included randomized controlled trials (ketamine vs midazolam).

| **Study ID** | **COUNTRY** | **STUDY  TYPE** | **INTERVENTION** | **CONTROL** | **SAMPLE SIZE  intervention comparator** | | **Study Population** | **MALE(%)  intervention comparator** | |
| --- | --- | --- | --- | --- | --- | --- | --- | --- | --- |
| Murrough 2015 | USA | RCT | 0.5mg\kg iv ketamine | 0.045mg\kg midazolam | 12 | 12 | Mood and anxiety spectrum disorders Patients | 33.3 | 33.3 |
| Pattanaseri 2024 | Thailand | RCT | 0.5mg\kg iv keteamine | 0.045mg\kg midazolam | 11 | 9 | Patients with treatment-resistant depression | 36.37 | 22.2 |
| Seraj 2025 | India | RCT | single oral dose (50mg\ml) | single oral dose(1mg\ml) | 40 | 40 | Adult inpatients with MDD and SI | 62.5 | 67.5 |
| Murrough 2013 | Multicenter | RCT | single iv infusion | single iv infusion | 47 | 25 | Patients with treatment-resistant Major depression | 45 | 56 |
| Tungpin 2023 | Taiwan | RCT | 0.5mg\kg iv infusion | 0,045mg\kg iv infusion | 42 | 42 | Patients with treatment-resistant depression | 33.3 | 26.2 |
| Price 2014 | USA | RCT | A single IV infusion of ketamine (0.5 mg/kg) | a single dose of midazolam 0.05 mg/kg | 36 | 21 | Patients with treatment-resistant unipolar major depression | 44 | 52 |
| Wei fan 2016 | China | RCT | Sub-anesthetic IV racemic ketamine (0.5 mg/kg) | Sub-anesthetic IV midazolam (0.05 mg/kg) | 20 | 17 | Cancer patients | NR | NR |
| Mark sinyor 2018 | Canada, US | RCT | Six IV ketamine infusions (0.5 mg/kg) | a single dose of midazolam 0.045 mg/kg | 9 | 4 | Adult inpatients with MDD | NR | NR |
| Feeney 2022 | USA | RCT | A single IV ketamine infusion (0.1, 0.5, or 1.0 mg/kg) | a single dose of midazolam 0.045 mg/kg | 40 | 16 | Subjects with DSM-IV-TR diagnosis of MDD | NR | NR |
| Hochschild 2021 | New york | RCT | Sub-anesthetic IV ketamine (0.5 mg/kg) | Sub-anesthetic IV midazolam (0.05 mg/kg) | 40 | 40 | Patients with DSM-IV MDD and clinically significant suicidal ideation | 45 | 35 |

Abbreviations: RCT, randomized controlled trial; IV, intravenous; MDD, major depressive disorder; SI, suicidal ideation; NR, not reported. Doses are reported as mg/kg unless otherwise specified.

**Supplementary Table 2**. Baseline participant characteristics of included randomized controlled trials.

| **Study ID** | **Age Mean SD** | | **BMI Mean SD** | | **MADRS Mean SD** | | **MADRS-SI Score Mean SD** | | **BSI Mean SD** | | **History of suicide attempt %** | | **Patient assessed total time** | |
| --- | --- | --- | --- | --- | --- | --- | --- | --- | --- | --- | --- | --- | --- | --- |
|  | intervention | comparator | intervention | comparator | intervention | comparator | Intervention | comparator | Intervention | Comparator | Intervention | Comparator | Intervention | Comparator |
| Murrough 2015 | 45.8(15.2) | 39.1(10.6) | NR | NR | 35.2(6.1) | 34.3(4.1) | NR | NR | 17.5(7.2) | 17.9(11.9) | 50 | 75 | 24 hrs | 24 hrs |
| Pattanaseri 2024 | 32.36(10.62) | 25.67(5.61) | 27.74(7.88) | 24.75(9.30) | 33.73(7.90) | 35.55(6.08) | NR | NR | NR | NR | NR | NR | one month | one month |
| Seraj 2025 | 33.1(10.9) | 35.6(12.5) | NR | NR | NR | NR | NR | NR | NR | NR | NR | NR | 7 days | 7 days |
| Murrough 2013 | 46.9(12.8) | 42.7(11.6) | 29.4(7.5) | 27.0(6.1) | 32.6(6.1) | 31.1(5.6) | NR | NR | NR | NR | 30 | 36 | 24 hrs | 24 hrs |
| Tungpin 2023 | 34.26(13.34) | 36.88(12.21) | 25.48(6.21) | 23.85(4.95) | 35.83(4.53) | 38.26(3.83) | NR | NR | NR | NR | 85.7 | 90.5 | 14 days | 14 days |
| Price 2014 | 48.6 (11.4) | 43.8 (10.9) | NR | NR | 33.3 (5.6) | 32.4 (4.8) | 1.61 (1.37) | 1.48 (1.03) | 6.1 (6.8) | 6.2 (6.7) | 28 | 38 | 24 hrs | 24 hrs |
| Fan 2016 | 46.75 (14.04) | 44.65 (15.1) | NR | NR | 34.89 ± 8.04 | 34.19 ± 10.83 | 3.65 ± 1.173 | 3.65 ± 1.268 | 17.06 ± 1.819 | 16.6 ± 2.137 | NR | NR | 7 days | 7 days |
| Mark sinyor 2018 | 36.80 (7.46) | 27.75 (3.77) | NR | NR | 42.20 ± 5.26 | 31.00 ± 9.02 | 2.2 ± 2.5 | 12.5 ± 15.0 | NR | NR | 60% | 100% | one month | one month |
| Feeney 2022 | 45.75(12.32) | 45.75(12.32) | NR | NR | NR | NR | 2.90 ± 0.74 | 2.69 ± 0.70 | NR | NR | NR | NR | one month | one month |
| Hochschild 2021 | 38.4 (13.2) | 40.7 (13.1) | NR | NR | NR | NR | 7.2 (6.4) | 13.3 (8.4) | NR | NR | NR | NR | 24 hrs | 24 hrs |

Values are mean (SD) unless stated otherwise. Abbreviations: BMI, body mass index; MADRS, Montgomery–Åsberg Depression Rating Scale; MADRS-SI, MADRS suicidal ideation item; BSS, Beck Scale for Suicide Ideation; NR, not reported.

| **Supplementary Table S3. GRADE Summary of Findings (ketamine vs midazolam).**  **Ketamine compared to Midazolam for Suicide** | | | | | | | | | | | |
| --- | --- | --- | --- | --- | --- | --- | --- | --- | --- | --- | --- |
| **Certainty assessment** | | | | | | | **Summary of findings** | | | | |
| **Participants (studies) Follow-up** | **Risk of bias** | **Inconsistency** | **Indirectness** | **Imprecision** | **Publication bias** | **Overall certainty of evidence** | **Study event rates (%)** | | **Relative effect (95% CI)** | **Anticipated absolute effects** | |
|  |  |  |  |  |  |  | **With Midazolam** | **With Ketamine** |  | **Risk with Midazolam** | **Risk difference with Ketamine** |
| **MADRS SI** | | | | | | | | | | | |
| **178 (4 RCTs)** | **not serious** | **serious^a^** | **not serious** | **not serious** | **none** | **⨁⨁⨁◯ Moderate^a^** | **73** | **105** | **-** | **73** | **MD 0.86 lower (1.42 lower to 0.29 lower)** |
| **BSS** | | | | | | | | | | | |
| **118 (3 RCTs)** | **not serious** | **serious^b^** | **not serious** | **not serious** | **none** | **⨁⨁⨁◯ Moderate^b^** | **50** | **68** | **-** | **50** | **MD 2.44 lower (5.15 lower to 0.28 higher)** |
| **MADRS** | | | | | | | | | | | |
| **84 (3 RCTs)** | **not serious** | **not serious** | **not serious** | **serious^c^** | **none** | **⨁⨁⨁◯ Moderate^c^** | **41** | **43** | **-** | **41** | **MD 6.23 lower (10.37 lower to 2.08 lower)** |
| **Nausea/Vomiting** | | | | | | | | | | | |
| **208 (4 RCTs)** | **not serious** | **serious^d^** | **not serious** | **not serious** | **none** | **⨁⨁⨁◯ Moderate^d^** | **23/103 (22.3%)** | **11/105 (10.5%)** | **OR 0.39 (0.17 to 0.87)** | **23/103 (22.3%)** | **122 fewer per 1,000 (from 177 fewer to 23 fewer)** |
| **Emotional Disturbance** | | | | | | | | | | | |
| **184 (3 RCTs)** | **not serious** | **not serious** | **not serious** | **serious^e^** | **none** | **⨁⨁⨁◯ Moderate^e^** | **0/91 (0.0%)** | **16/93 (17.2%)** | **OR 12.30 (2.20 to 68.74)** | **0/91 (0.0%)** | **0 fewer per 1,000 (from 0 fewer to 0 fewer)** |
| **Derealization** | | | | | | | | | | | |
| **236 (3 RCTs)** | **not serious** | **not serious** | **not serious** | **not serious** | **none** | **⨁⨁⨁⨁ High** | **7/107 (6.5%)** | **44/129 (34.1%)** | **OR 11.71 (4.62 to 29.64)** | **7/107 (6.5%)** | **385 more per 1,000 (from 179 more to 609 more)** |
| **Dizziness** | | | | | | | | | | | |
| **176 (3 RCTs)** | **not serious** | **serious^f^** | **not serious** | **not serious** | **none** | **⨁⨁⨁◯ Moderate^f^** | **15/77 (19.5%)** | **34/99 (34.3%)** | **OR 1.97 (0.95 to 4.08)** | **15/77 (19.5%)** | **128 more per 1,000 (from 8 fewer to 302 more)** |

**CI:** confidence interval; **MD:** mean difference; **OR:** odds ratio

**Explanations**

a. Heterogeneity I = 63% before sensitivity Analysis

b. Heterogeneity I = 64% before sensitivity Analysis

c. The confidence interval is very wide, and the sample size is insufficient to produce a precise estimate.

d. Heterogeneity I = 59% before sensitivity Analysis

e. The estimate is based on limited data, and the confidence interval is wide, indicating high uncertainty.

f. Heterogeneity I = 57% before sensitivity Analysis

**Before and after sensitivity analysis Forest plots**

**MADR SI Score**


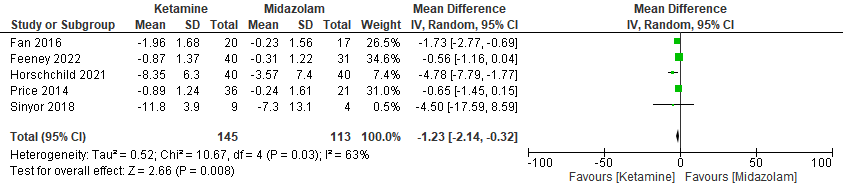

Figure 3. MADR SI before sensitivity analysis


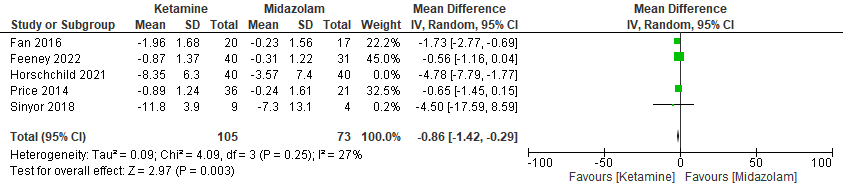

Figure 4. MADR SI after sensitivity analysis excluding Horschchild 2021

**BSS**


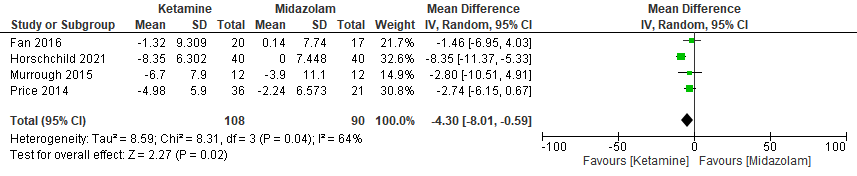

Figure 5. BSS before sensitivity analysis


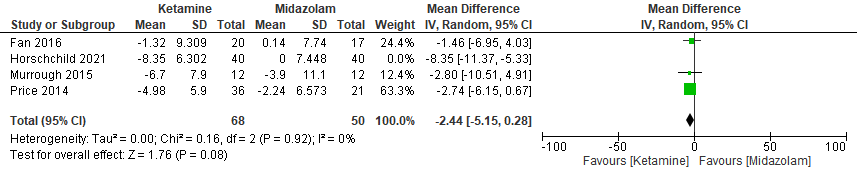

Figure 6. BSS after sensitivity analysis excluding Horschchild 2021

**NAUSEA/Vomiting**


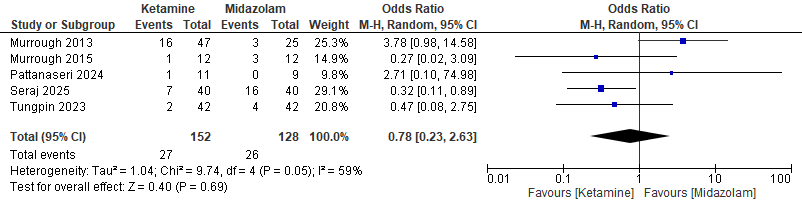

Figure 7. Nausea before sensitivity analysis


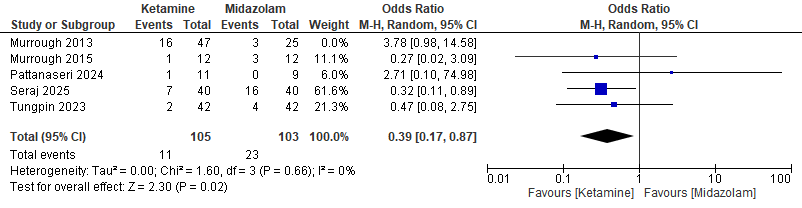

Figure 8. Nausea after sensitivity analysis excluding Murrough 2013 was

**Dizziness**


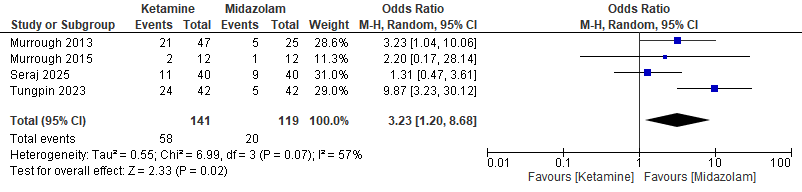

Figure 9. Dizziness before sensitivity analysis


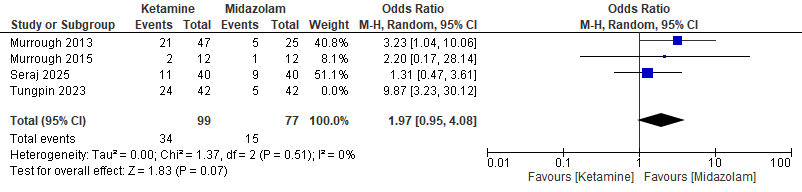

Figure 10. Dizziness after sensitivity analysis excluding Tungpin 2023 was not included)
